# Supplementary material for: Synergistic Antitumor Effects of Caerin Peptides and Dendritic Cell Vaccines in a 4T-1 Murine Breast Cancer Model
Source: Vaccines (Basel). 2025 May 28;13(6):577. doi: 10.3390/vaccines13060577 (PMC12197375; doi:10.3390/vaccines13060577)
Supplement: Supplementary file 1 [file vaccines-13-00577-s001.zip › vaccines-3581023-supplementary.pdf]

A

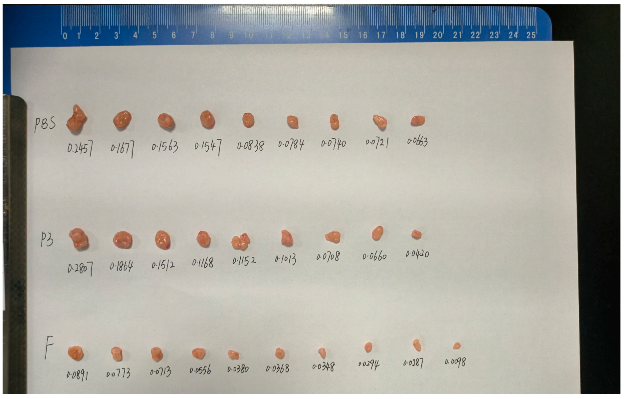

B

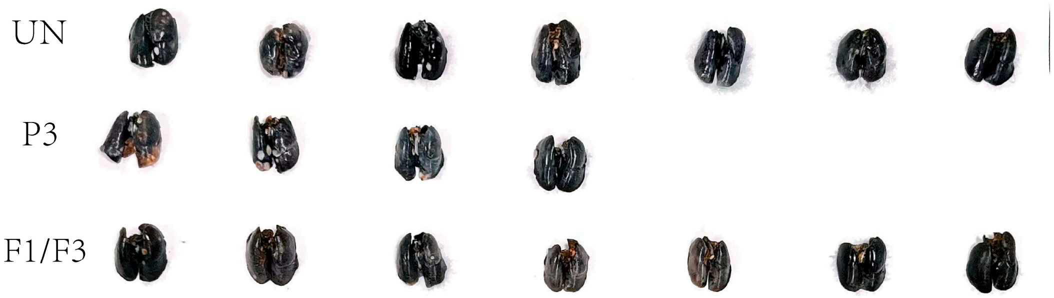

Figure S1. F1/F3 Inhibits 4T-1 Tumor Growth In Vivo.

Mice bearing flank tumors received PBS, P3, or F1/F3. On Day 18, the tumors were excised and weighed ***(A)****. In a separate experiment, tumors were implanted in the fourth mammary fat pad, and after PBS, P3, or F1/F3 treatment, lungs were harvested on Day 30 and stained with 15% India ink (due to the lengthy experimental period, the P3 group exhibited significant mortality, reducing the number of available lung samples)* ***(B)****. Each group included 3–8 mice. Panels* ***(A–B)*** *represent one independent experiment, repeated once.*

Table S1. Mouse Lung Metastatic Nodules.

UN group		P3 group		F1/F3 group	
Number of mice	Nodule numbers	Number of mice	Nodule numbers	Number of mice	Nodule numbers
Mouse ①	11	Mouse ①	36	Mouse ①	3
Mouse ②	7	Mouse ②	18	Mouse ②	3
Mouse ③	7	Mouse ③	12	Mouse ③	2
Mouse ④	4	Mouse ④	7	Mouse ④	1
Mouse ⑤	4	Mouse ⑤	5	Mouse ⑤	0
Mouse ⑥	3	Mouse ⑥	3	Mouse ⑥	6
Mouse ⑦	0	Mouse ⑦	2	Mouse ⑦	6
Mouse ⑧	17	Mouse ⑧	1	Mouse ⑧	3
Mouse ⑨	17	Mouse ⑨	26	Mouse ⑨	1
Mouse ⑩	16	Mouse ⑩	23	Mouse ⑩	0
Mouse ⑪	11	Mouse ⑪	3	Mouse ⑪	0
Mouse ⑫	5	Mouse ⑫	1	Mouse ⑫	0
Mouse ⑬	2				
Mouse ⑭	0				
Average number of nodules	7	Average number of nodules	11	Average number of nodules	2

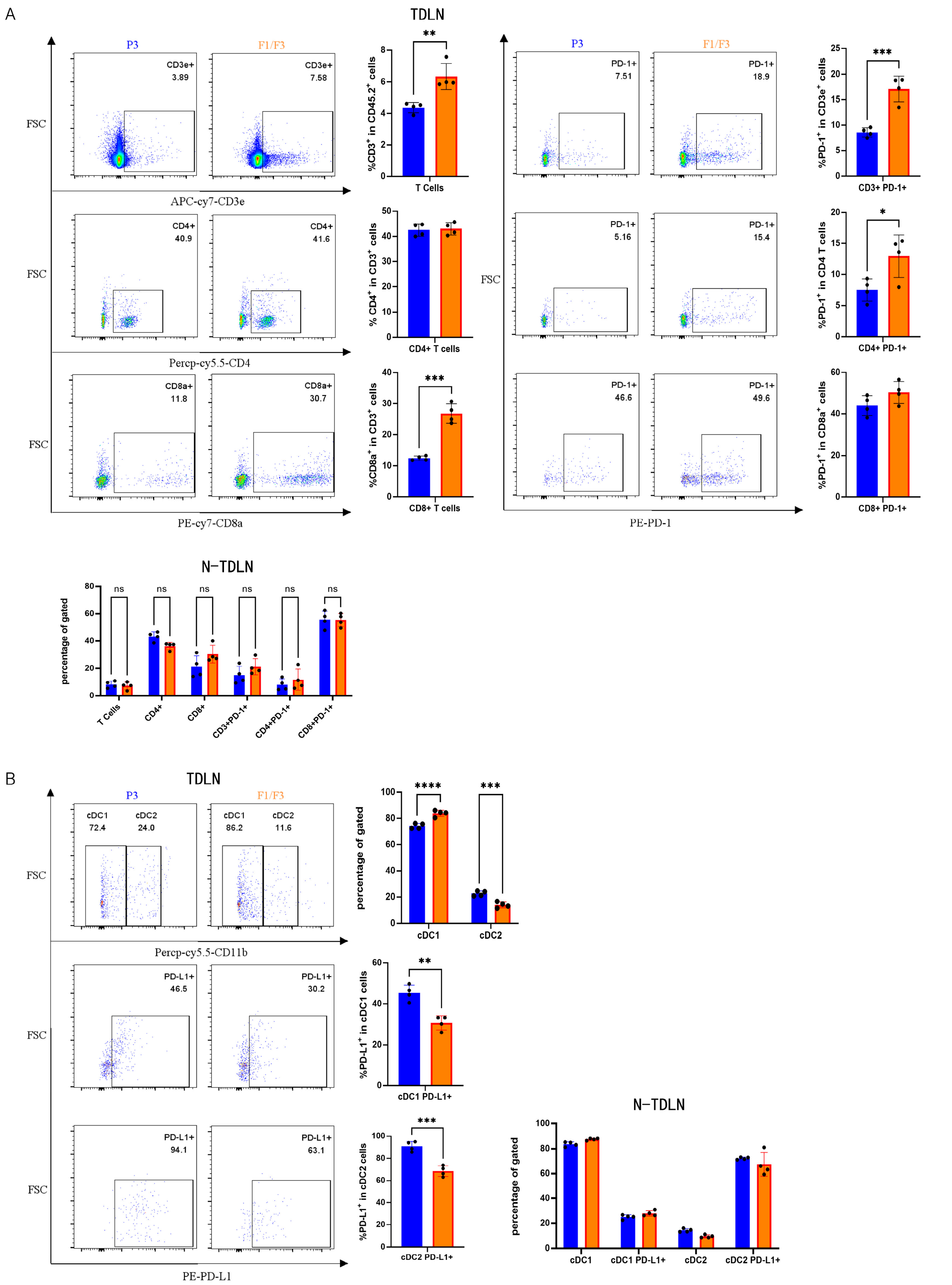

Figure S2. F1/F3 Improves the Lymph Node Immune Profile in B16 Tumor–Bearing Mice.

B16 cells were inoculated in mice, and draining/non-draining lymph nodes from P3- and F1/F3-treated groups were analyzed by flow cytometry to evaluate immune cells, DC subsets, and related markers, including: ***(A)*** *T cells (CD45.2$^+$CD3e$^+$), CD4$^+$ T cells (CD45$^+$CD3e$^+$CD4$^+$), CD8$^+$ T cells (CD45$^+$CD3e$^+$CD8$^+$), PD-1 expression on CD4$^+$ T cells (CD45$^+$CD3e$^+$CD4$^+$PD-1$^+$), PD-1 expression on CD8$^+$ T cells (CD45$^+$CD3e$^+$CD8$^+$PD-1$^+$);* ***(B)*** *cDC1 (CD45.2$^+$Lineage$^-$CD11c$^+$MHCII$^+$CD11b$^-$), cDC2 (CD45.2$^+$Lineage$^-$CD11c$^+$MHCII$^+$CD11b$^+$), PD-L1 (CD45.2$^+$Lineage$^-$CD11c$^+$MHCII$^+$PD-*

L1$^+$), PD-1 (CD45.2$^+$CD3e$^+$PD-1$^+$), PD-L1 on cDC1 (CD45.2$^+$Lineage$^-$CD11c$^+$MHCII$^+$CD11b$^-$PD-L1$^+$), PD-L1 on cDC2 (CD45.2$^+$Lineage$^-$CD11c$^+$MHCII$^+$CD11b$^+$PD-L1$^+$). Each experiment included 3–6 mice per group. Panels ***(A–B)*** *represent a single independent experiment, repeated at least once. Data are shown as mean ± SD. ns: not significant; **P < 0.05; **P < 0.01; ***P < 0.001; ****P < 0.0001*. Student's t-test was performed* ***(A–B)****.*

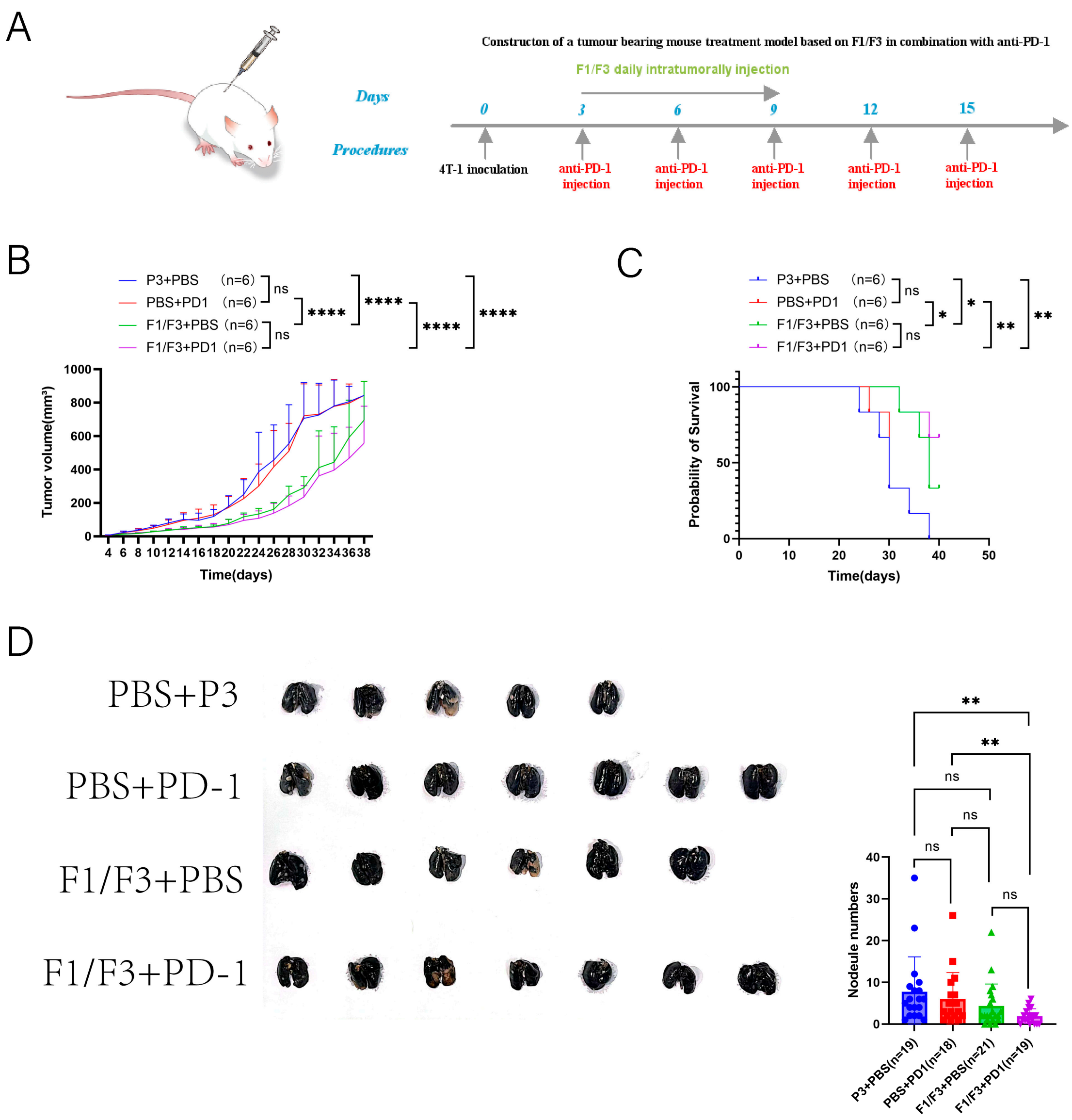

Figure S3. No Significant Difference in 4T-1 Tumor Progression between F1/F3 and F1/F3 + anti-PD-1.

***(A)** Mice bearing tumors in the fourth mammary fat pad received PBS, P3, F1/F3, or F1/F3 + anti-PD-1 treatments; **(B)** Tumor size and **(C)** survival rates were assessed; **(D)** On Day 30, lungs were harvested, stained with 15% India ink, and the number of tumor nodules was recorded. Each group contained 3–8 mice. Panels **(A–C)** are from one independent experiment, while **(D)** compiles results from three independent experiments. All were repeated once. Data are shown as mean ± SD. ns: not significant; *P < 0.05; **P < 0.01. Two-way ANOVA was applied to **(B)**, Kaplan–Meier survival to **(C)**, and Student's t-test to **(D)**.*

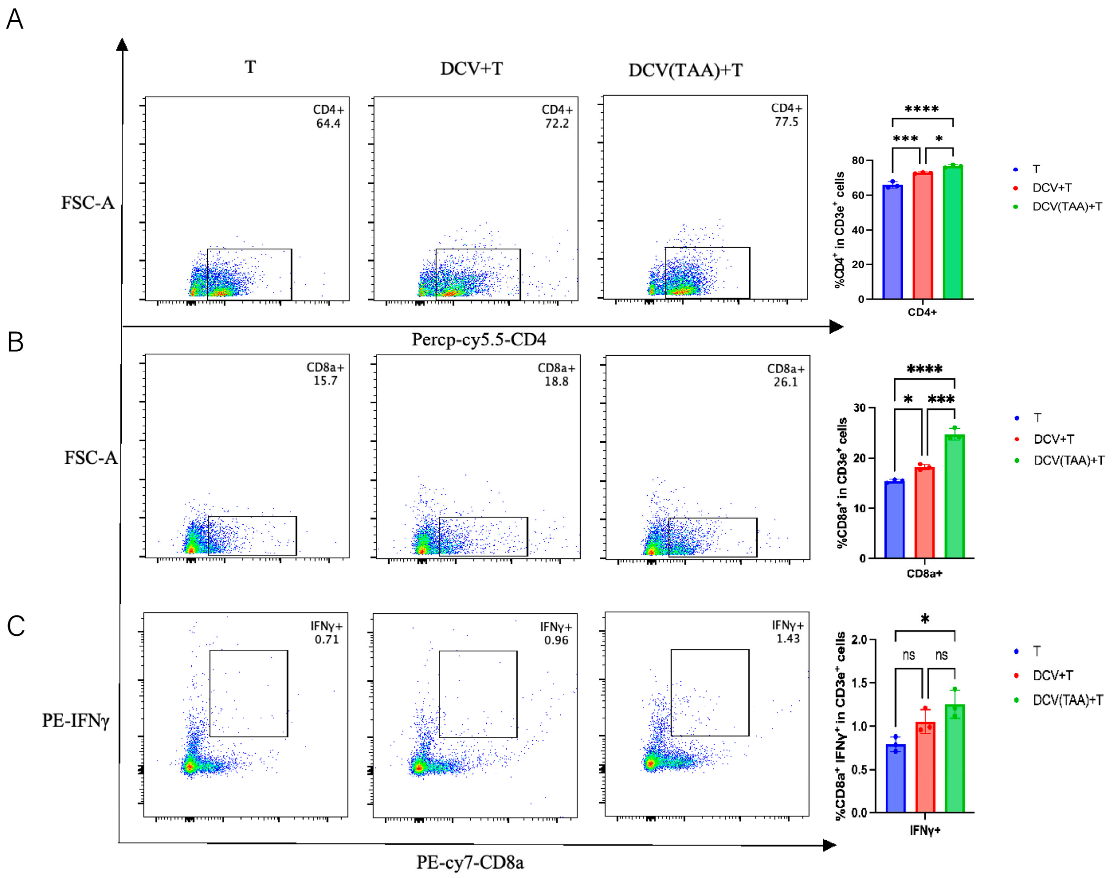

Figure S4. Full Antigen–Loaded DC Vaccine More Effectively Stimulates T-Cell Proliferation and Function.

Flow cytometry was used to detect ***(A)*** *$CD4^+$ T cells ($CD45.2^+CD3^+CD4^+$) and* ***(B)*** *$CD8^+$ T cells ($CD45.2^+CD3^+CD8^+$);* ***(C)*** *IFN-γ ($CD45.2^+CD3^+CD8^+IFN\text{-}\gamma^+$). Panels* ***(A–C)*** *each represent one independent experiment, repeated once. Data are shown as mean ± SD. ns: not significant; *$P < 0.05$; ***$P < 0.001$; ****$P < 0.0001$. One-way ANOVA was performed for* ***(A–C)****.*

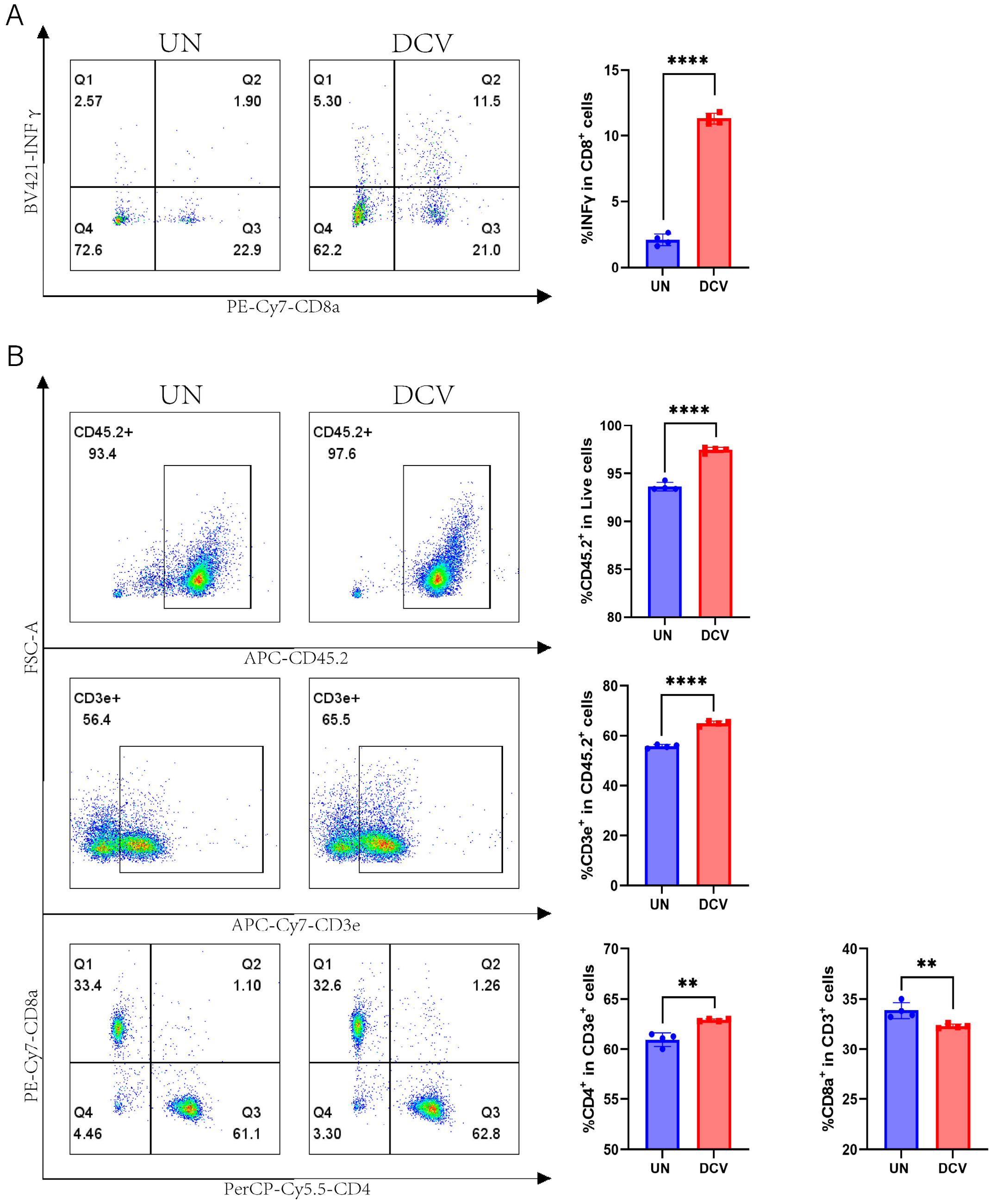

Figure S5. Flow Cytometric Analysis of Immune Cell Changes in Non-Draining Lymph Nodes of UN vs. DCV Groups.

(A) *INF-γ (CD45$^+$CD3e$^+$CD8$^+$INF-γ$^+$) secretion and T-cell subsets, including* ***(B)*** *CD45.2$^+$ cells, T cells (CD45.2$^+$CD3e$^+$), CD4$^+$ T cells (CD45$^+$CD3e$^+$CD4$^+$), CD8$^+$ T cells (CD45$^+$CD3e$^+$CD8$^+$). Each group included 3–6 mice. Panels* ***(A–B)*** *each represent a single independent experiment, repeated once. Values are shown as mean ± SD. ns: not significant; **P < 0.01; ****P < 0.0001. A t-test was used for* ***(A–B)***.

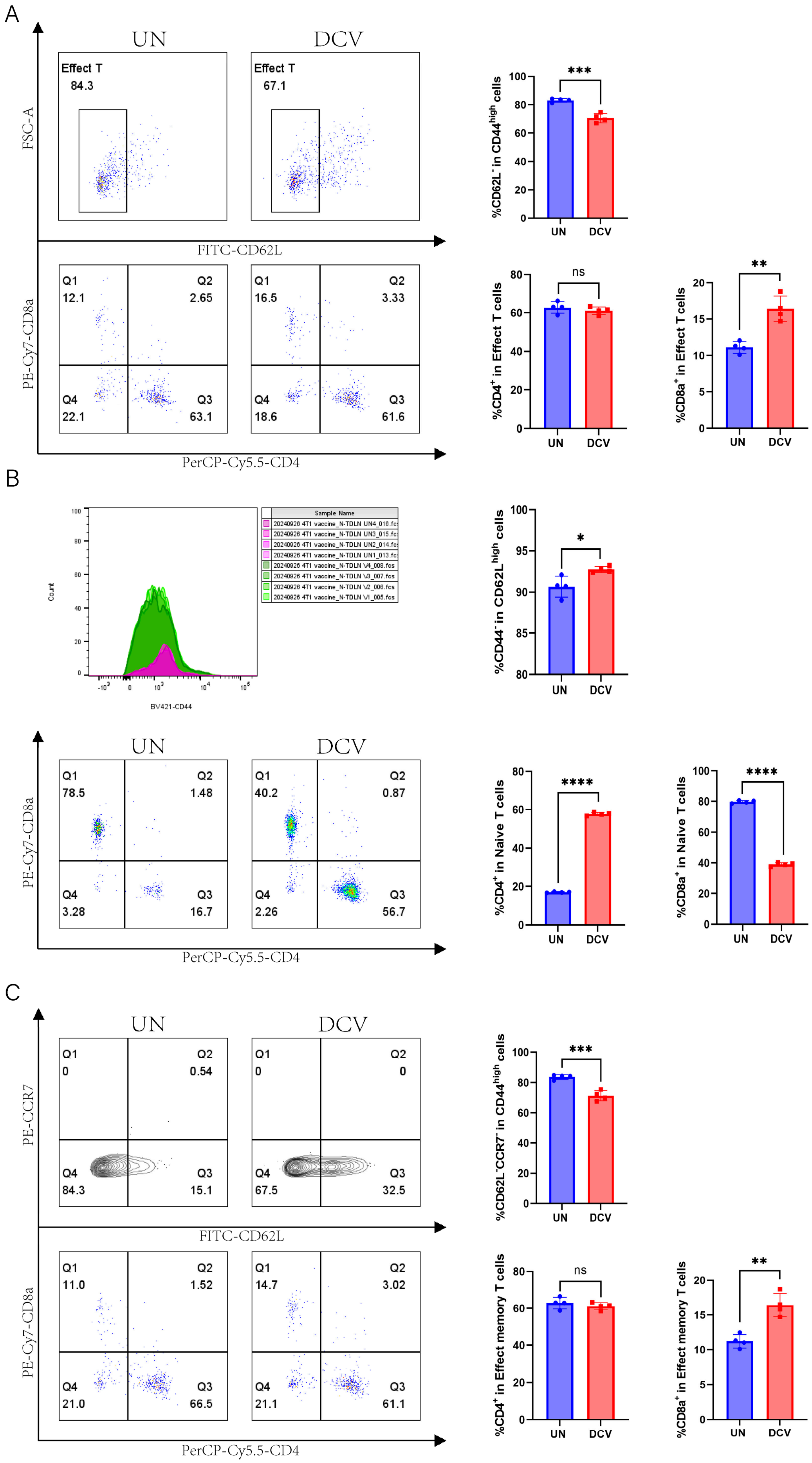

A
UN
DCV
Effect T
84.3
67.1
FSC-A
FITC-CD62L
PE-Cy7-CD8a
PerCP-Cy5.5-CD4
Q1 12.1
Q2 2.65
Q3 63.1
Q4 22.1
Q1 16.5
Q2 3.33
Q3 61.6
Q4 18.6
%CD62L⁻ in CD44high cells
%CD4+ in Effect T cells
%CD8a+ in Effect T cells
***
ns
**
B
Sample Name
Count
BV421-CD44
%CD44⁻ in CD62L high cells
*
Q1 78.5
Q2 1.48
Q3 16.7
Q4 3.28
Q1 40.2
Q2 0.87
Q3 56.7
Q4 2.26
%CD4+ in Naive T cells
%CD8a+ in Naive T cells
****
C
PE-CCR7
Q1 0
Q2 0.54
Q3 15.1
Q4 84.3
Q1 0
Q2 0
Q3 32.5
Q4 67.5
%CD62L⁻CCR7⁻ in CD44high cells
Q1 11.0
Q2 1.52
Q3 66.5
Q4 21.0
Q1 14.7
Q2 3.02
Q3 61.1
Q4 21.1
%CD4+ in Effect memory T cells
%CD8a+ in Effect memory T cells

Figure S6. Flow Cytometric Analysis of T Cell Subsets in Non-Draining Lymph Nodes: UN Group vs. DCV Group.

(A) *effector T cells (CD45.2⁺CD3e⁺CD44^highCD62L-), effector CD4⁺ T cells (CD45.2⁺CD3e⁺CD44highCD62L-CD4⁺), effector CD8⁺ T cells (CD45.2⁺CD3e⁺CD44highCD62L-CD8⁺);* ***(B)*** *naïve T cells (CD45.2⁺CD3e⁺CD62LhighCD44-), naïve CD4⁺ T cells (CD45.2⁺CD3e⁺CD62LhighCD44-CD4⁺), naïve CD8⁺ T cells (CD45.2⁺CD3e⁺CD62LhighCD44-CD8⁺);* ***(C)*** *effector memory T cells (CD45.2⁺CD3e⁺CD44highCD62L-CCR7-), effector memory CD4⁺ T (CD45.2⁺CD3e⁺CD44highCD62L-CCR7-CD4⁺), effector memory CD8⁺ T (CD45.2⁺CD3e⁺CD44highCD62L-CCR7-CD8⁺). Each group included 3–6 mice. Panels* ***(A–C)*** *each represent a single independent experiment, repeated once. Values are shown as mean ± SD. ns: not significant; *P < 0.05; **P < 0.01; ***P < 0.001; ****P < 0.0001. A t-test was used for* ***(A–C)****.*

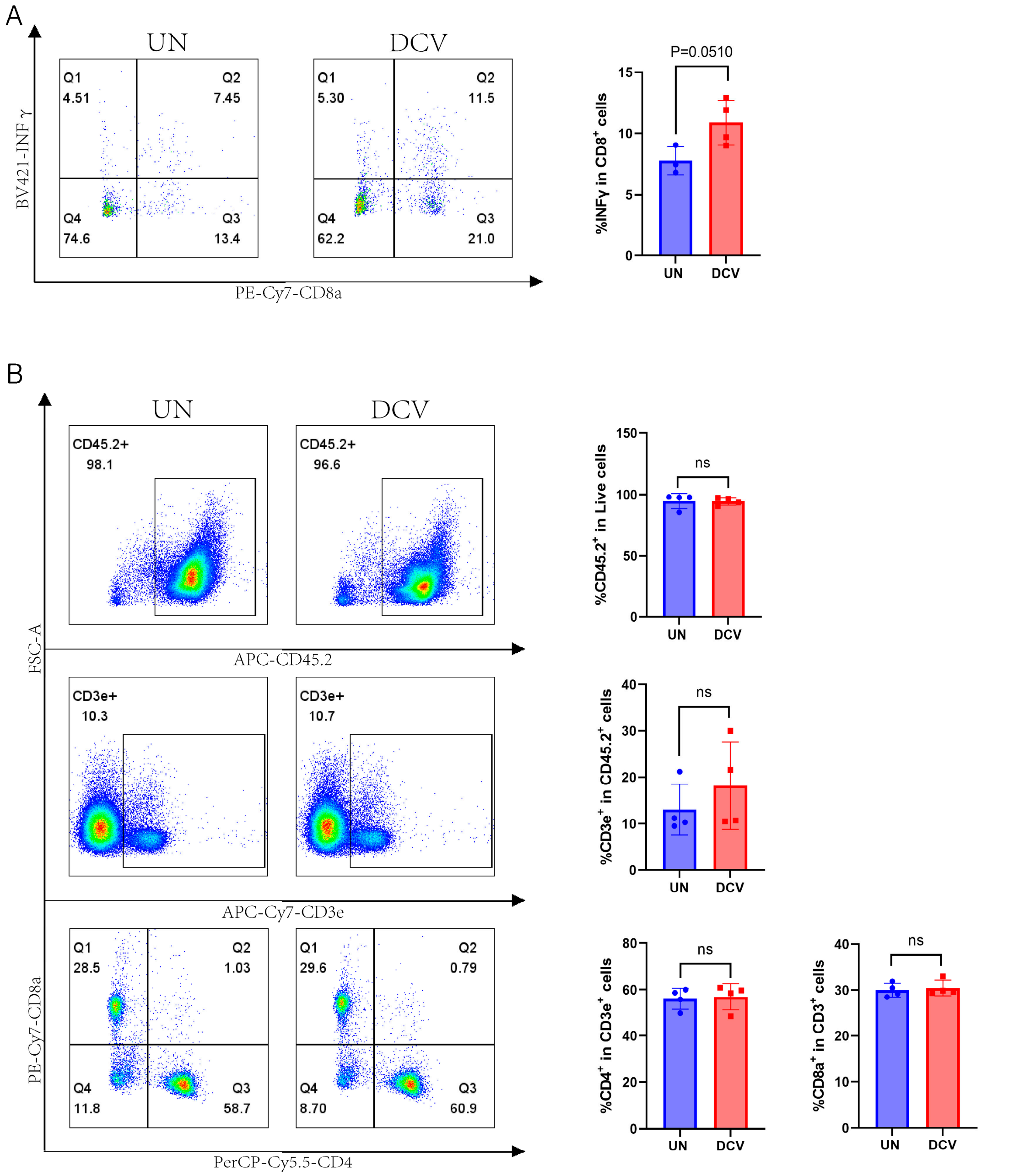

Figure S7. Flow Cytometric Analysis of Immune Cell Changes in the Spleens of UN vs. DCV Groups.

(A) *INF-γ (CD45^{+}CD3e^{+}CD8^{+}INF-γ^{+}) secretion and T-cell subsets, including* ***(B)*** *CD45.2^{+} cells, T cells (CD45.2^{+}CD3e^{+}), CD4^{+} T cells (CD45^{+}CD3e^{+}CD4^{+}), CD8^{+} T cells (CD45^{+}CD3e^{+}CD8^{+}. Each group included 3–6 mice. Panels* ***(A–B)*** *each represent a single independent experiment, repeated once. Data are shown as mean ± SD. ns: not significant. A t-test was used for* ***(A–B)****.*

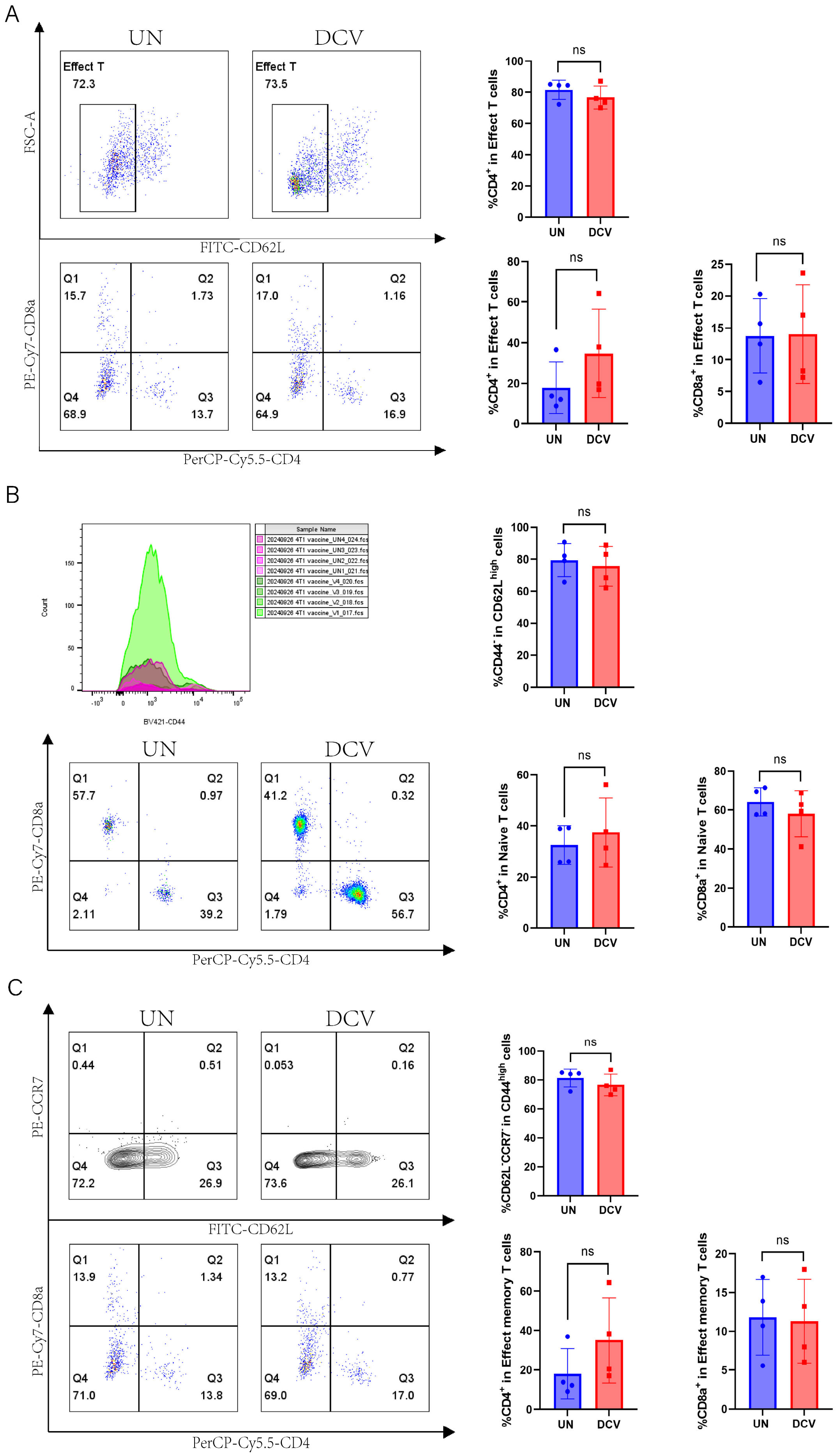
A
UN
DCV
Effect T
72.3
Effect T
73.5
FSC-A
FITC-CD62L
Q1 15.7
Q2 1.73
Q4 68.9
Q3 13.7
Q1 17.0
Q2 1.16
Q4 64.9
Q3 16.9
PE-Cy7-CD8a
PerCP-Cy5.5-CD4
%CD4+ in Effect T cells
%CD8a+ in Effect T cells
ns
UN
DCV
B
Sample Name
Count
BV421-CD44
UN
DCV
Q1 57.7
Q2 0.97
Q4 2.11
Q3 39.2
Q1 41.2
Q2 0.32
Q4 1.79
Q3 56.7
PE-Cy7-CD8a
PerCP-Cy5.5-CD4
%CD44- in CD62L high cells
%CD4+ in Naive T cells
%CD8a+ in Naive T cells
ns
C
UN
DCV
Q1 0.44
Q2 0.51
Q4 72.2
Q3 26.9
Q1 0.053
Q2 0.16
Q4 73.6
Q3 26.1
PE-CCR7
FITC-CD62L
Q1 13.9
Q2 1.34
Q4 71.0
Q3 13.8
Q1 13.2
Q2 0.77
Q4 69.0
Q3 17.0
PE-Cy7-CD8a
PerCP-Cy5.5-CD4
%CD62L-CCR7- in CD44high cells
%CD4+ in Effect memory T cells
%CD8a+ in Effect memory T cells
ns

Figure S8. Flow Cytometric Analysis of T Cell Subsets Changes in the Spleens of UN vs. DCV Groups.

(A) *effector T cells (CD45.2^{+}CD3e^{+}CD44^highCD62L^{-}), effector CD4^{+} T cells (CD45.2^{+}CD3e^{+}CD44^{high}CD62L^{-}CD4^{+}), effector CD8^{+} T cells (CD45.2^{+}CD3e^{+}CD44^{high}CD62L^{-}CD8^{+});* ***(B)*** *naïve T cells (CD45.2^{+}CD3e^{+}CD62L^{high}CD44^{-}), naïve CD4^{+} T cells (CD45.2^{+}CD3e^{+}CD62L^{high}CD44^{-}CD4^{+}), naïve CD8^{+} T cells (CD45.2^{+}CD3e^{+}CD62L^{high}CD44^{-}CD8^{+});* ***(C)*** *effector memory T cells (CD45.2^{+}CD3e^{+}CD44^{high}CD62L^{-}CCR7^{-}), effector memory CD4^{+} T (CD45.2^{+}CD3e^{+}CD44^{high}CD62L^{-}CCR7^{-}CD4^{+}), effector memory CD8^{+} T (CD45.2^{+}CD3e^{+}CD44^{high}CD62L^{-}CCR7^{-}CD8^{+}). Each group included 3–6 mice. Panels* ***(A–C)*** *each represent a single independent experiment, repeated once. Data are shown as mean ± SD. ns: not significant. A t-test was used for* ***(A–C)****.*

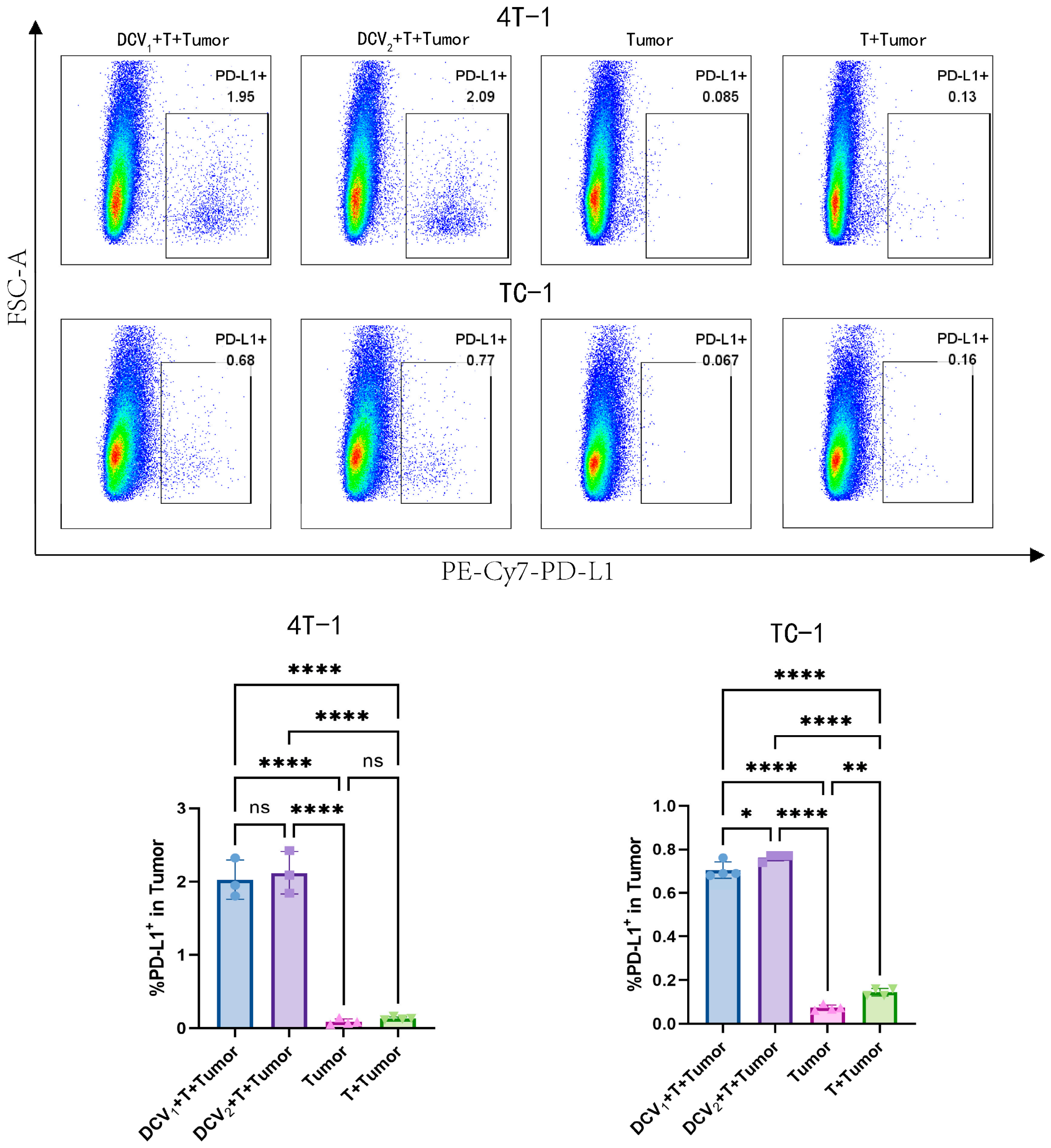

Figure S9. Flow Cytometric Analysis of PD-L1 Expression in Co-Cultures of DC, T, and Tumor. *Tumor cells co-cultured with DCV_1 or DCV_2 and T cells displayed a marked increase in PD-L1 on tumor cells. Data represent a single independent experiment, repeated once, shown as mean ± SD. ns: not significant; *P < 0.05; **P < 0.01; ****P < 0.0001. A t-test was used for statistical analysis.*

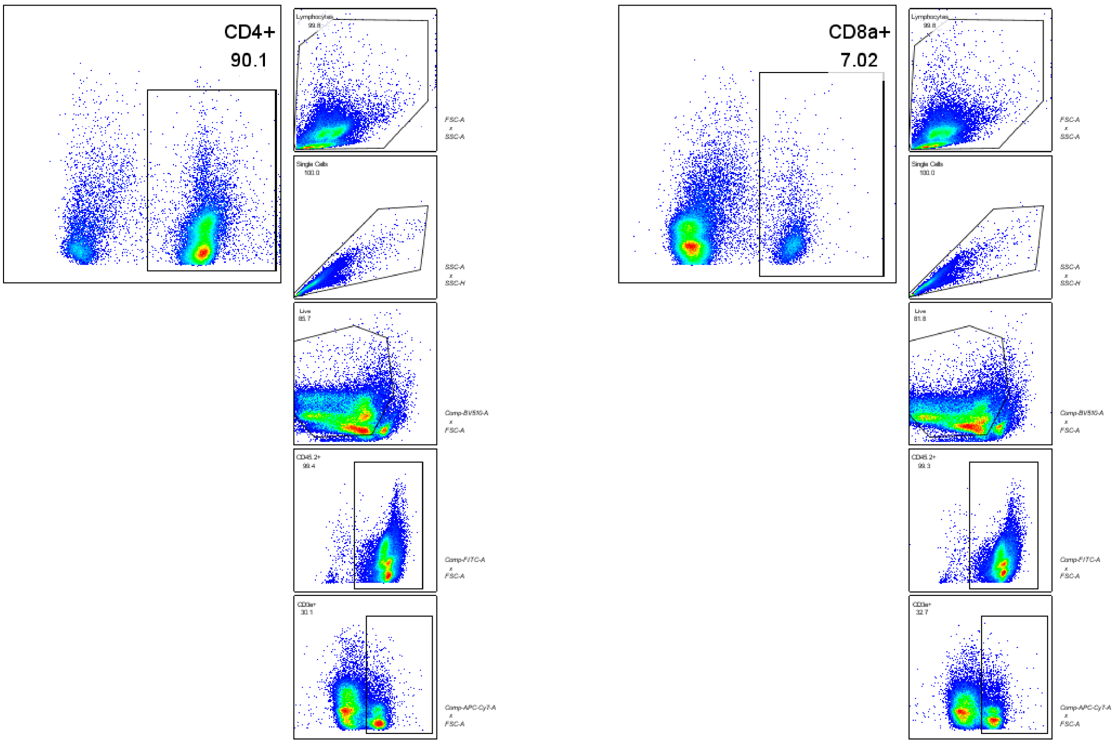

Figure S10. Gating Strategy for Flow Cytometric Analysis of $CD4^{+}$/$CD8^{+}$ T Cells in 4T1 Tumor Microenvironment (TME).

T cells ($CD45.2^{+}CD3e^{+}$); $CD4^{+}$ T cells ($CD45^{+}CD3e^{+}CD4^{+}$); $CD8^{+}$ T cells ($CD45^{+}CD3e^{+}CD8^{+}$).

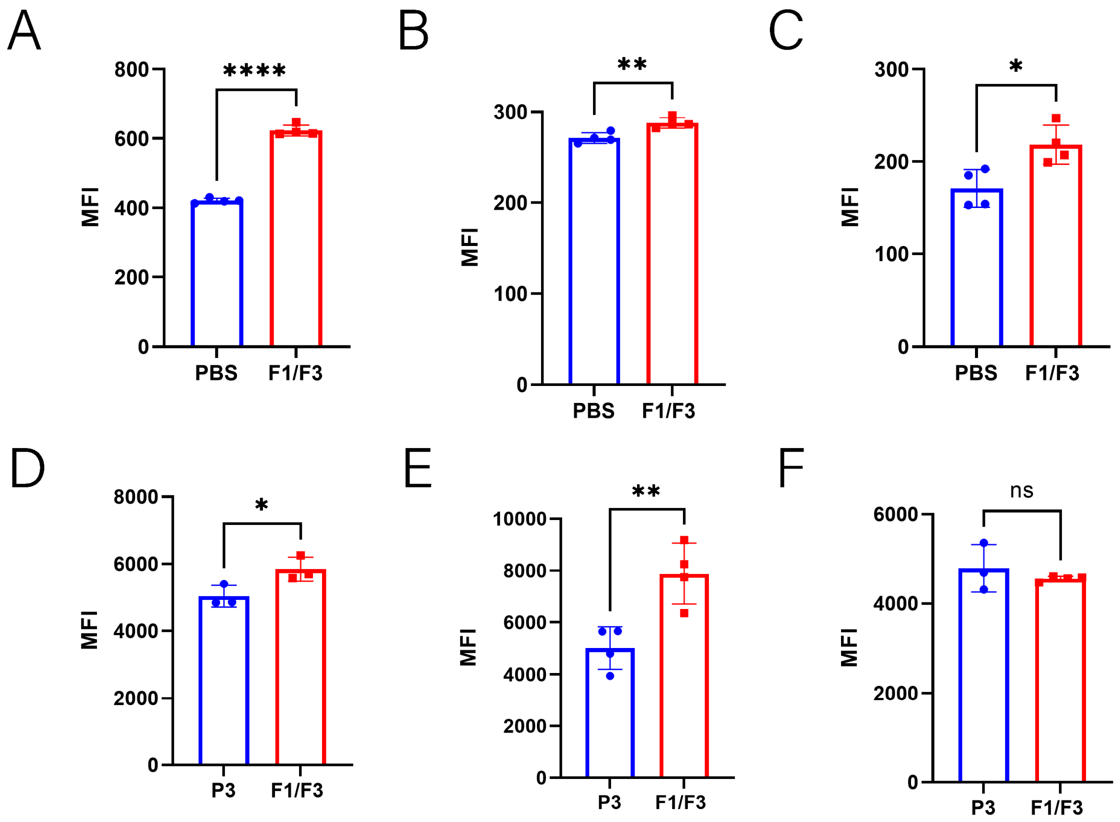

Figure S11. Flow cytometry analysis of MFI values for PD-1 expression on immune cells. *Tumor-draining lymph nodes (TDLNs) in the 4T-1 model **(A)** $CD3^+PD\text{-}1^+$; Non-tumor-draining lymph nodes (non-TDLNs) in the 4T-1 model **(B)** $CD4^+PD\text{-}1^+$, **(C)** $CD8^+PD\text{-}1^+$; Tumor-draining lymph nodes (TDLNs) in the B16 model **(D)** $CD3^+PD\text{-}1^+$;**(E)** $CD4^+PD\text{-}1^+$; **(F)** $CD8^+PD\text{-}1^+$. Data are shown as mean ± SD. ns: not significant; *$P < 0.05$;**$P < 0.01$; ****$P < 0.0001$. A t-test was used for **(A–F)**.*
